# Global prevalence of autoimmune diseases in turner syndrome: a systematic review and meta-analysis

**DOI:** 10.1080/07853890.2025.2573143

**Published:** 2025-11-16

**Authors:** Seongbeen Hwang, Yonghee Park, Hyunoh Moon, Kihun Kim, Sukdong Yoo, Su-Yeon Cho, Yujin Kwon, Won Kyu Kim, Yun Hak Kim

**Affiliations:** ^a^ School of Medicine, Pusan National University, Yangsan, Republic of Korea; ^b^ Department of Occupational and Environmental Medicine, Pusan National University Yangsan Hospital, Yangsan, Republic of Korea; ^c^ Department of Pediatrics, School of Medicine, Pusan National University Yangsan Hospital, Yangsan, Republic of Korea; ^d^ Research Institute for Convergence of Biomedical Science and Technology, Pusan National University Yangsan Hospital, Yangsan, Republic of Korea; ^e^ Natural Product Research Center, Korea Institute of Science and Technology (KIST), Gangneung, Republic of Korea; ^f^ Natural Product Applied Science, KIST School, University of Science and Technology (UST), Gangneung, Republic of Korea; ^g^ Department of Convergence Medicine, Yonsei University Wonju College of Medicine, Wonju, Republic of Korea; ^h^ Department of Biomedical Informatics, School of Medicine, Pusan National University, Yangsan, Republic of Korea; ^i^ Department of Anatomy, School of Medicine, Pusan National University, Yangsan, Republic of Korea; ^j^ Research Institute for Convergence of Biomedical Science and Technology, Pusan National University Yangsan Hospital, Pusan, Republic of Korea

**Keywords:** Turner syndrome, autoimmune diseases, sex chromosome abnormalities, prevalence, systematic review, meta-analysis

## Abstract

**Objective:**

Despite previous research linking Turner Syndrome (TS) with specific autoimmune conditions, a comprehensive analysis examining the broader relationship between sex chromosome abnormalities and multiple autoimmune diseases (AIDs) is lacking. This study aims to provide a meta-analysis of AID prevalence in TS, highlighting clinical implications and potential genetic links.

**Methods:**

A systematic search of Medline, Embase, Scopus, and Web of Science was conducted from database inception to July 30, 2024, using search terms including “Turner Syndrome,” “sex chromosome abnormalities,” and “autoimmune diseases.” Duplicate entries were removed, and three authors independently screened the titles and abstracts, resolving discrepancies through consensus. Eligible studies included case-control, cohort, and cross-sectional designs that assessed the prevalence of autoimmune diseases in patients with sex chromosome abnormalities. Studies were excluded if they lacked relevant data or focused on unrelated genetic conditions. Meta-analyses were performed using Review Manager (version 5.4.1).

**Results:**

A total of 2,430 records were identified, and 1,215 duplicate records were removed by an automation tool. The remaining 1,215 records were screened for relevance, resulting in 535 records that were assessed based on titles and abstracts. Ultimately, 45 studies met the inclusion criteria for systematic review and meta-analysis, comprising 14,717 patients with TS. The pooled prevalence of AIT in TS patients was 21.61% (95% confidence interval: 12.85–30.37). Compared to the general population, the prevalence rates were elevated for T1DM (1.32%), celiac disease (5.89%), and alopecia areata (0.84%) in TS patients. Conversely, the prevalence of psoriasis (1.14%) did not differ significantly, whereas the prevalence of vitiligo (0.84%) was significantly lower.

**Conclusions:**

This meta-analysis demonstrates a significantly higher prevalence of certain autoimmune conditions in TS patients relative to the general population. These findings also suggest potential genetic associations between AIDs and the X chromosome, highlighting avenues for further investigation.

## KEY MESSAGES

- Patients with Turner Syndrome (TS) exhibit a significantly higher prevalence of autoimmune diseases (AIDs), including Autoimmune Thyroiditis (AIT), Type 1 Diabetes Mellitus (T1DM), Crohn’s disease, celiac disease, and alopecia areata, compared to the general population.
- Among thyroid disorders, Hashimoto’s Thyroiditis was particularly prevalent in TS patients, whereas Graves’ Disease showed a prevalence similar to the general population.
- Given the increased risk of autoimmune diseases in TS, regular monitoring, early diagnosis, and personalized healthcare strategies are essential for improving patient outcomes and mitigating long-term complications.

## Introduction

Approximately 1 in 400 male and 1 in 650 female live births exhibit some form of sex chromosome abnormality [1]. While the symptoms of these conditions are generally less severe than those linked to autosomal abnormalities, they can lead to a range of complications, including physical, cardiovascular, endocrine, renal, developmental, and psychosocial issues [1]. Turner Syndrome (TS), which affects females and is characterized by the complete or partial absence of one X chromosome, is one of the most common sex chromosome abnormalities, with a prevalence of about 1 in 2,500 individuals [2]. Another frequent abnormality is Klinefelter Syndrome (KS), which affects males and caused by an extra X chromosome (47, XXY) [3]. Both conditions present various medical challenges that require thorough medical evaluation and care.

Notably, autoimmune diseases (AIDs) demonstrate marked sex differences in prevalence, with a strong female predominance for most conditions [4–7]. These differences are thought to result from a combination of genetic, hormonal, and environmental factors, all of which contribute to immune system regulation [8]. Given that TS is a female-specific condition, this sex-related predisposition to AIDs is particularly relevant. Indeed, women generally exhibit a higher prevalence of AIDs such as autoimmune thyroiditis, rheumatoid arthritis (RA), systemic lupus erythematosus (SLE), and Sjögren’s syndrome [9]. The skewed sex ratio in AID prevalence suggests that sex chromosomes and related hormonal influences play important roles in modulating autoimmunity [4,5,10]. Furthermore, X chromosome dosage and inactivation patterns have been implicated as possible mechanisms underlying the increased autoimmune susceptibility observed in females and in conditions like TS [11,12]

Although previous meta-analyses have established significant correlations between TS and conditions such as celiac disease [13], Crohn’s disease [14], and thyroid disease [15], these studies have focused solely on specific disorders in patients with TS. There remains a lack of comprehensive reviews and meta-analyses that examine the broader relationship between sex chromosome abnormalities and AIDs. While we initially included a range of sex chromosome abnormalities in our search terms, only one study focused on KS, leading us to rely primarily on prevalence data from studies centered on TS. Therefore, this study aims to explore the association between Turner syndrome and multiple AIDs, emphasizing the clinical implications and potential health outcomes of these relationships.

## Methods

### Eligibility criteria

The study adhered to the PRISMA guidelines, and the PRISMA checklist is provided in the Supporting Information [16]. As this study is a systematic review and meta-analysis based on previously published data, ethical approval was not required. This systematic review and meta-analysis included data from case-control, cohort, and cross-sectional studies that investigated the prevalence of autoimmune diseases in individuals with sex chromosome abnormalities. Additionally, conference papers, abstracts were also included. To be eligible, studies had to feature an exposure group consisting of participants with sex chromosome abnormalities. The included AIDs considered include AIT, T1DM, alopecia areata, vitiligo, Crohn’s disease, lupus, psoriasis, and lichen planus, among others. Studies such as experimental reviews, case reports, or any research not providing relevant data on the association between sex chromosome abnormalities and AIDs were excluded. Additionally, papers focusing on conditions unrelated to our topic, such as autosomal chromosome deletion, skewed X-inactivation, and mosaicism, were also excluded.

### Search strategy and data sources

Relevant studies were systematically searched across electronic databases, including MEDLINE (1966 to July 30, 2024), Embase (1947 to July 30, 2024), Scopus (1966 to July 30, 2024), and Web of Science (1900 to July 30, 2024). The search strategies incorporated both Medical Subject Headings (MeSH) terms and free-text keywords related to sex chromosome abnormalities and AIDs. The detailed search strategy is provided in Supplementary Table 1. We reviewed the reference lists of selected articles and identified additional studies through a manual search using Google and Google Scholar. No language restrictions were applied.

### Selection criteria

Three authors (SB, YH, HO) conducted the initial database search, independently screened for duplicate entries, and assessed the titles and abstracts of studies related to sex chromosome abnormalities and AIDs. During the selection process, duplicate records were initially detected and removed using the duplicate removal function within the EndNote program. Any remaining duplicates not automatically detected by EndNote were further identified and removed manually by the authors during the screening of titles and abstracts. They then reviewed the full-text articles to determine eligibility for inclusion in the analysis. Any discrepancies during the selection process were discussed and resolved by consensus among the authors.

### Data extraction

Data extraction was performed independently by three authors (SB, YH, HO) using a standardized data extraction form. The extracted information included the author’s name, year of publication, study type, country, study period, total sample size, patient sample size, disease type, and disease diagnostic criteria. Any discrepancies between the authors were resolved through discussion and consensus.

The risk of bias in each study was qualitatively assessed using the Newcastle-Ottawa Scale (NOS) for cohort and case-control studies [17]. For cross-sectional studies, we used an adapted version of the NOS [18]. This approach enabled us to critically evaluate the risk of bias in the included studies and assess the overall quality of the evidence [19]. The risk of bias for each included study was independently assessed by three authors (SB, YH, HO). Any discrepancies between the authors were resolved through discussion and consensus.

### Publication bias

All analyses of publication bias were conducted using R version 4.4.1. Publication bias for the main results was assessed visually with Doi plots and quantitatively with the Luis-Kanamori (LFK) index [20]. These tools are widely recognized for evaluating publication bias, particularly when prevalence is the primary effect measure [21]. An LFK index score of ±1 suggests ‘no asymmetry,’ scores between ±1 and ±2 indicate ‘minor asymmetry,’ and scores of ±2 or more indicate ‘major asymmetry’ [22]. The Egger test and funnel plot were also conducted to assess potential publication bias. The trim-and-fill method was used to estimate the results, accounting for studies that might have been missed due to publication bias [23].

### Statistical analysis

Our primary analysis focused on overall prevalence, with subgroup analyses conducted for specific AIDs, including AIT, T1DM, alopecia areata, vitiligo, Crohn’s disease, lupus, psoriasis, and lichen planus, among others. The prevalence of AIDs was calculated as the total number of cases divided by the total number of subjects. Pooled prevalences were determined using the generic inverse variance method [24]. In studies where the standard error (SE) was not reported, it was calculated using the following formula:

SE=√(p(1−p)/n)×100,95%confidence interval (CI)=p±1.96×SE wherep=prevalence

We assessed heterogeneity between studies using the I^2^ statistic [25], and where heterogeneity exceeds 50%, a random-effects model was employed [26,27]. All analyses were conducted using Review Manager (version 5.4.1). We also evaluated the association between TS and the risk of various AIDs, accounting for potential confounding factors.

## Results

### Study selection and characteristics

**Figure 1.**
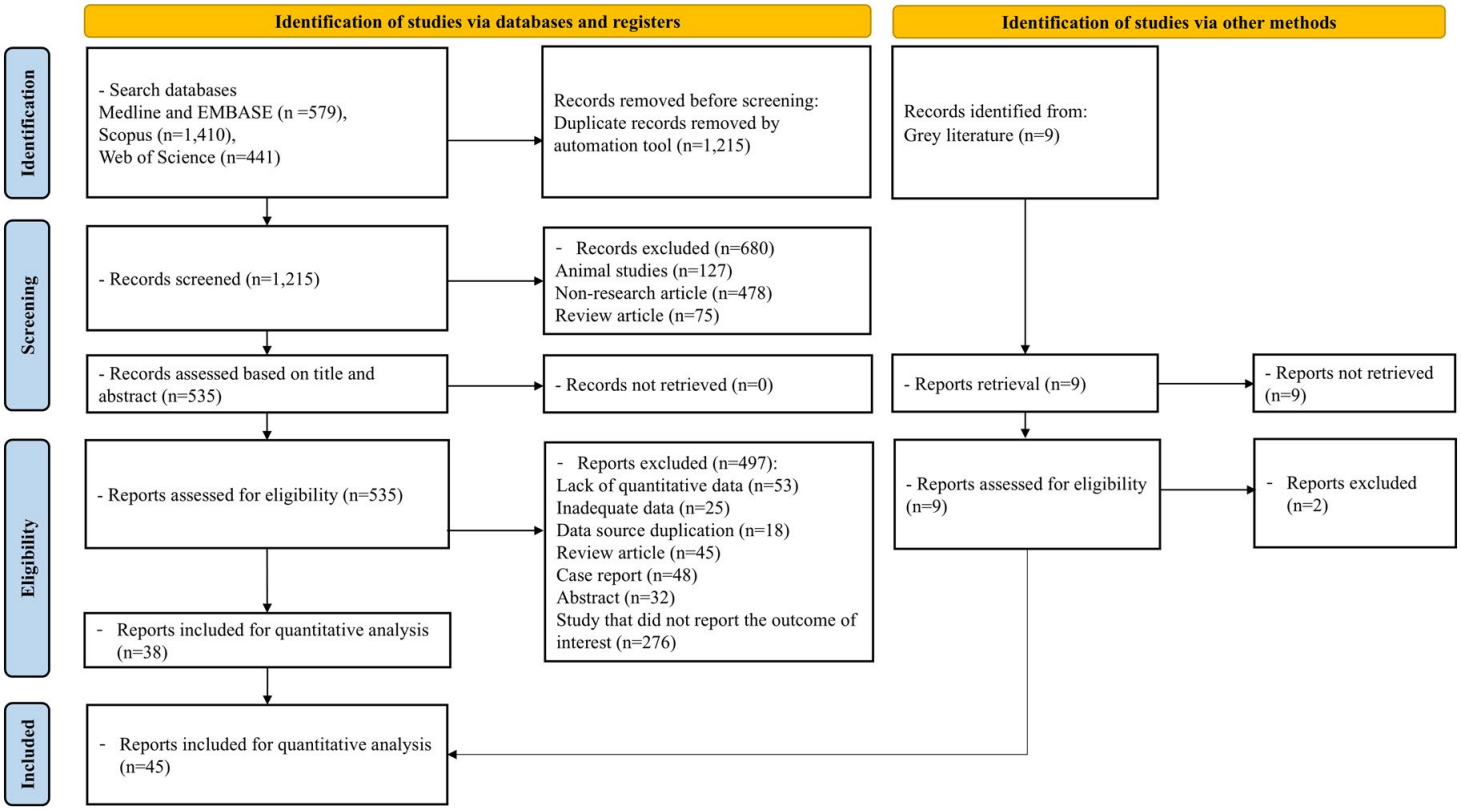
Flow diagram illustrating the selection process for included studies in systematic review and meta-analysis.

From the initial search, a total of 1,215 records were obtained after removing duplicates. After excluding non-research articles, animal studies, and review articles, 535 studies were screened by reviewing their titles and abstracts. Studies were excluded for reasons such as lack of quantitative data, insufficient data, data duplication, being review articles, case reports, or abstracts. Following full-text review, 38 studies were selected for inclusion. An additional 7 studies were identified through manual searches and the reference lists of included articles, bringing the total to 45 studies in our meta-analysis, consisting of 1 cohort study, 2 case-control studies, and 42 cross-sectional studies [28–72]. The PRISMA flowchart is shown in Figure 1. Although our initial search included all sex chromosome abnormalities, the limited availability of studies on abnormalities other than TS, with only one such study [73], led us to focus our meta-analysis exclusively on prevalence data from TS studies. Information on the included studies is presented in Table 1. These studies, published between 1998 and 2024, span 21 countries, and analyze a total of 14,717 patients with TS.

**Table 1.** Characteristics of included studies.

Author	Year	Study type	Country	Study period	N of total sample	N of patient sample	Disease type	Disease Diagnostic criteria
Bonamico [44] M.	1998	Cross-sectional	Italy	Unclear	37	3	Celiac disease	Biopsy
Gravholt CH. [42]	1998	Cross-sectional	Denmark	1/1984-12/1993	594	1	Psoriasis	ICD-8
Gravholt CH. [42]	1998	Cross-sectional	Denmark	1/1984-12/1993	594	9	Type1 diabetes mellitus	ICD-8
Ivarsson SA. [45]	1999	Cross-sectional	Sweden	Unclear	87	4	Celiac disease	Biopsy
Gillett PM. [46]	2000	Cross-sectional	Canada	01/12/1998-01/10/1999	45	1	Celiac disease	Biopsy
Rujner J. [47]	2001	Cross-sectional	Poland	Unclear	48	2	Celiac disease	Biopsy
Bonamico M. [48]	2002	Cross-sectional	Italy	Unclear	389	25	Celiac disease	Biopsy
Sakly W. [49]	2005	Cross-sectional	France	Unclear	47	7	Celiac disease	Sero anti t-TG Abs or AEA positive
Bahremand HMSH. [50]	2005	Cross-sectional	Iran	01/10/2002-2004	48	2	Celiac disease	Biopsy
Bettendorf M. [51]	2006	Cross-sectional	Germany	Unclear	120	5	Celiac disease	Sero anti t-TG Abs or AEA positive
Ságodi L. [52]	2006	Cross-sectional	Unclear	1994–2003	63	5	Celiac disease	Biopsy
Stenberg AE. [70]	2007	Cross-sectional	Sweden	Unclear	97	4	Celiac disease	Medical records
Hirschfield GM. [61]	2008	Cross-sectional	Canada	Unclear	63	4	Celiac disease	Biopsy
Mortensen KH. [53]	2009	Cross-sectional	Denmark	Unclear	106	5	Celiac disease	Biopsy
Frost AR. [54]	2009	Cross-sectional	London	Unclear	256	12	Celiac disease	Biopsy
Fukada I.	2009	Cross-sectional	Japan	Unclear	65	20	Hashimoto’s thyroiditis	TSH, FT4 levels and TGAb, TPOAb
Fukada I.	2009	Cross-sectional	Japan	Unclear	65	3	Grave’s disease	Medical records
Dias Mdo C. [55]	2010	Cross-sectional	Brazil	Unclear	56	2	Celiac disease	Biopsy
Nabhan ZM. [56]	2011	Cross-sectional	U.S.A.	2000–2010	77	4	Celiac disease	Sero anti t-TG Abs or AEA positive
Freriks K. [57]	2011	Cross-sectional	Netherland	05/2005-06/2009	150	3	Celiac disease	Sero anti t-TG Abs or AEA positive
Kammoun M. [65]	2012	Cross-sectional	Tunisia	01/2007-12/2011	37	2	Celiac disease	Unclear
Bakalov VK. [40]	2012	Cross-sectional	U.S.A.	1/2000-3/2009	224	6	Celiac disease	Medical records
Bakalov VK. [40]	2012	Cross-sectional	U.S.A.	1/2000-3/2009	224	1	Vitiligo	Medical records
Bakalov VK. [40]	2012	Cross-sectional	U.S.A.	1/2000-3/2009	224	6	Grave’s disease	Medical records
Bakalov VK. [40]	2012	Cross-sectional	U.S.A.	1/2000-3/2009	224	2	Type1 diabetes mellitus	Medical records
Bakalov VK. [40]	2012	Cross-sectional	U.S.A.	1/2000-3/2009	224	7	Psoriasis	Medical records
Bakalov VK. [40]	2012	Cross-sectional	U.S.A.	1/2000-3/2009	224	82	Hashimoto’s thyroiditis	Medical records
Bakalov VK. [40]	2012	Cross-sectional	U.S.A.	1/2000-3/2009	224	1	Alopecia areata	Medical records
Nadeem M. [58]	2013	Cross-sectional	Ireland	Unclear	32	3	Celiac disease	Biopsy
Hamza RT. [39]	2013	Cross-sectional	Egypt	10/2009-11/2010	80	5	Celiac disease	Sero anti t-TG Abs or AEA positive and biopsy
Hamza RT. [39]	2013	Cross-sectional	Egypt	10/2009-11/2010	80	1	Type1 diabetes mellitus	Unclear
Grossi A. [34]	2013	Cross-sectional	Italy	Unclear	66	26	Autoimmune thyroiditis	TSH, FT4 levels and TGAb, TPOAb
Grossi A. [34]	2013	Cross-sectional	Italy	Unclear	66	14	Hashimoto’s thyroiditis	TSH, FT4 levels and TGAb, TPOAb
Goldacre MJ. [59]	2014	Cross-sectional	England	1999–2011	2,459	45	Celiac disease	Medical records
Bessahraoui M NM. [67]	2014	Cross-sectional	Algeria	2007–2013	33	4	Celiac disease	Unclear
Stagi S. [64]	2014	Cohort	Italy	06/2003-05/2011	32	3	Celiac disease	Biopsy
Valenzise M. [38]	2014	Cross-sectional	Italy	Unclear	408	7	Grave’s disease	Laboratory test
Rutigliano I. [60]	2015	Cross-sectional	Italy	Unclear	31	4	Celiac disease	Medical records
Yeşilkaya E. [33]	2015	Cross-sectional	Turkey	9/2013-2/2014	714	18	Celiac disease	Medical records
Yeşilkaya E. [33]	2015	Cross-sectional	Turkey	9/2013-2/2014	842	5	Alopecia areata	Medical records
Yeşilkaya E. [33]	2015	Cross-sectional	Turkey	9/2013-2/2014	842	6	Vitiligo	Medical records
Yeşilkaya E. [33]	2015	Cross-sectional	Turkey	9/2013-2/2014	842	10	Psoriasis	Medical records
Mårild K. [71]	2016	Case-control	Sweden	1997–2006	5	1	Celiac disease	Biopsy
Larizza D. [37]	2016	Cross-sectional	Italy	1980–2014	87	5	Celiac disease	Unclear
Larizza D. [37]	2016	Cross-sectional	Italy	1980–2014	87	1	Crohn’s disease	Unclear
Larizza D. [37]	2016	Cross-sectional	Italy	1980–2014	87	23	Autoimmune thyroiditis	Unclear
Witkowska-Sedek E. [72]	2017	Cross-sectional	Poland	1990–2002	14	5	Autoimmune thyroiditis	Medical records
Baz Ouidad SM. [63]	2018	Cross-sectional	Algeria	2015–2017	85	12	Celiac disease	Biopsy
Dumitrescua C GI. [68]	2018	Cross-sectional	Romania	Unclear	93	3	Celiac disease	Unclear
Elechi H. [69]	2018	Cross-sectional	England	2008–2017	28	1	Celiac disease	Medical records
Gawlik AM. [62]	2018	Case-control	Poland	Unclear	37	3	Celiac disease	Unclear
Hanew K. [36]	2018	Cross-sectional	Japan	08/1993-08/2009	385	3	Crohn’s disease	Self-report
Hanew K. [36]	2018	Cross-sectional	Japan	08/1993-08/2009	424	107	Autoimmune thyroiditis	Self-report
Berglund A. [66]	2019	Cross-sectional	Denmark	2003–2008	141	2	Celiac disease	Biopsy
Wegiel M. [35]	2019	Cross-sectional	Poland	2001–2018	73	2	Celiac disease	Sero anti t-TG Abs or AEA positive and biopsy
Wegiel M. [35]	2019	Cross-sectional	Poland	2001–2018	134	3	Vitiligo	Medical records
Wegiel M. [35]	2019	Cross-sectional	Poland	2001–2018	134	2	Psoriasis	Medical records
Wegiel M. [35]	2019	Cross-sectional	Poland	2001–2018	134	2	Type1 diabetes mellitus	Hyperglycemia and positive serology
Wegiel M. [35]	2019	Cross-sectional	Poland	2001–2018	134	20	Hashimoto’s thyroiditis	TSH, FT4 levels and TGAb, TPOAb
Farquhar M. [32]	2020	Cross-sectional	Canada	2/2015-07/2018	122	11	Celiac disease	Medical records
Abdel-Badie Salem N. [31]	2021	Cross-sectional	Egypt	03/2019-03/2020	55	2	Celiac disease	Sero anti t-TG Abs or AEA positive and biopsy
Abdel-Badie Salem N. [31]	2021	Cross-sectional	Egypt	03/2019-03/2020	55	4	Autoimmune thyroiditis	Unclear
Abdel-Badie Salem N. [31]	2021	Cross-sectional	Egypt	03/2019-03/2020	55	1	Type1 diabetes mellitus	Unclear
Abdel-Badie Salem N. [31]	2021	Cross-sectional	Egypt	03/2019-03/2020	55	1	Psoriasis	Unclear
Said JT. [30]	2022	Cross-sectional	U.S.A.	06/2010-12/2019	236	7	Alopecia areata	Medical records
Said JT. [30]	2022	Cross-sectional	U.S.A.	06/2010-12/2019	236	5	Vitiligo	Medical records
Witkowska-Krawczak E. [29]	2023	Cross-sectional	Poland	1990–2002	39	2	Celiac disease	Medical records
Witkowska-Krawczak E. [29]	2023	Cross-sectional	Poland	1990–2002	45	9	Autoimmune thyroiditis	Medical records
Lee YL. [43]	2023	Cross-sectional	Malaysia	08/2020-07/2022	96	8	Autoimmune thyroiditis	TSH, FT4 levels and TGAb, TPOAb
Naessén S. [28]	2024	Cross-sectional	Sweden	1994–2020	502	19	Celiac disease	Medical records

# tTG Abs, antibodies against tissue transglutaminase; AEA, anti-endomysium; TSH, Thyroid-stimulating hormone; FT4, Free thyroxine test; TPOAb, thyroid peroxide antibody; ICD, International Statistical Classification of Diseases and Related Health Problems.

### Synthesis of results

**Figure 2.**
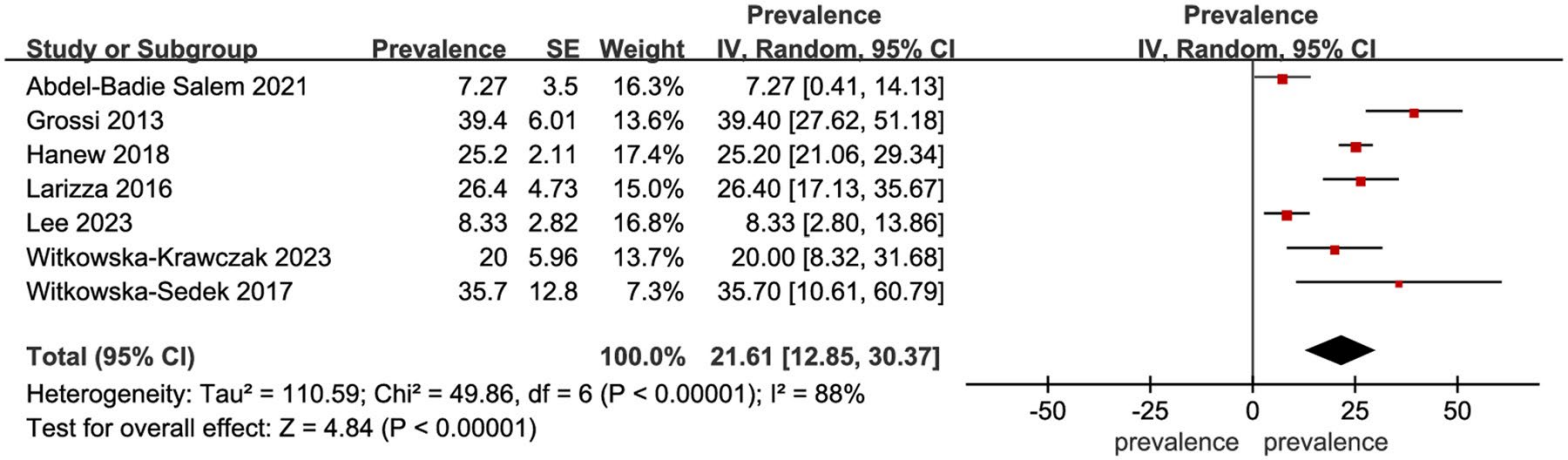
Forest plot showing the prevalence of autoimmune thyroiditis in Turner syndrome.

Seven studies involving 787 TS patients were used to evaluate the prevalence of AIT, with a pooled prevalence of 21.61% (95% CI: 12.85–30.37, I^2^ = 88%, *p* < 0.001) (Figure 2). Four studies involving 489 TS patients were included in assessing the prevalence of Hashimoto’s Thyroiditis (HT), with a pooled prevalence of 25.78% (95% CI: 14.29–37.28, I^2^ = 88%, *p* < 0.001) (Supplementary Figure 1). Three studies with 697 TS patients evaluated Graves’ disease (GD), showing a pooled prevalence of 2.08% (95% CI: 1.03–3.14, I^2^ = 0%, *p* < 0.001) (Supplementary Figure 2).

Five studies involving 1,087 TS patients assessed the prevalence of T1DM, resulting in a pooled prevalence of 1.32% (95% CI: 0.64–2.00, I^2^ = 0%, *p* = 0.0001) (Supplementary Figure 3). Two studies of 472 TS patients evaluated Crohn’s disease, with a pooled prevalence of 1.13% (95% CI: 0.92–1.34, I^2^ = 0%, *p* < 0.001) (Supplementary Figure 4).

**Figure 3.**
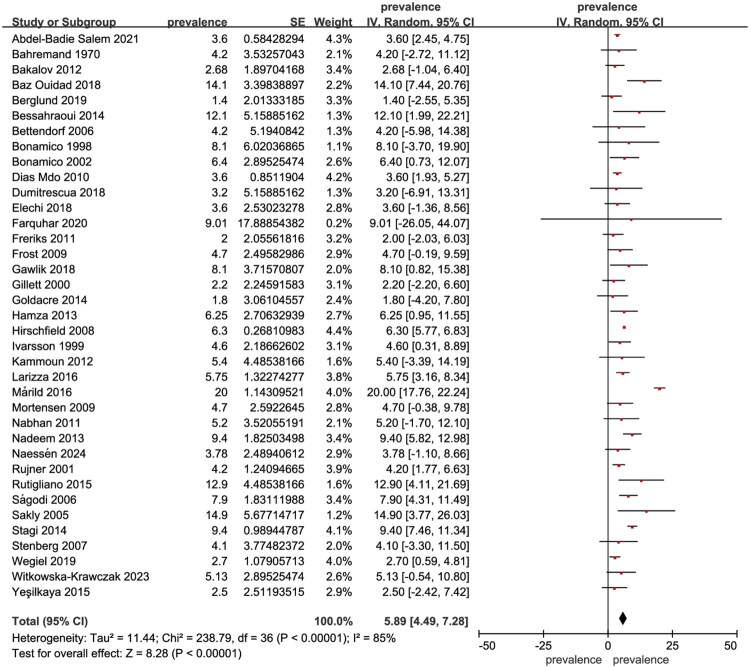
Forest plot showing the prevalence of celiac disease in Turner syndrome.

Thirty-eight studies involving 6,598 TS patients evaluated celiac disease, with a pooled prevalence of 5.89% (95% CI: 4.49–7.28, I^2^ = 85%, *p* < 0.001) (Figure 3). Three studies including 1,302 TS patients assessed alopecia areata, showing a pooled prevalence of 0.84% (95% CI: −0.02–1.70, I^2^ = 58%, *p* = 0.05) (Supplementary Figure 5).

Four studies involving 1,436 TS patients assessed vitiligo, with a pooled prevalence of 0.84% (95% CI: 0.24–1.44, I^2^ = 23%, *p* = 0.006) (Supplementary Figure 6). Lastly, five studies with 1,849 TS patients reported a pooled prevalence of psoriasis of 1.14% (95% CI: 0.18–2.11, I^2^ = 70%, *p* = 0.02) (Supplementary Figure 7). The overall results are summarized in Table 2.

**Table 2.** Summary of pooled prevalence of autoimmune disease in turner syndrome.

Disease	Number of included studies	Number of total TS patients	Number of TS patients with AIDs	Pooled prevalence
Autoimmune thyroiditis	7	787	182	21.61% (95% CI: 12.85–30.37)
Hashimoto’s thyroiditis	4	489	136	25.78% (95% CI: 14.29–37.28)
Grave’s disease	3	697	16	2.08% (95% CI: 1.03–3.14)
Type 1 Diabetes Mellitus	5	1087	15	1.32% (95% CI: 0.64–2.00)
Crohn’s disease	2	472	4	1.13% (95% CI: 0.92–1.34)
Celiac disease	38	6,598	240	5.89% (95% CI: 4.49–7.28)
Alopecia Areata	3	1,302	13	0.84% (95% CI: −0.02 − 1.70)
Vitiligo	4	1,436	15	0.84% (95% CI: 0.24–1.44)
Psoriasis	5	1,849	21	1.14% (95% CI: 0.18–2.11)

# CI, Confidence Interval; I^2^, a measure assessing the percentage of between-study variation due to differences in prevalence estimates across studies rather than chance.

### Risk of bias in individual studies

Among the 41 cross-sectional studies, 11 were rated as “good” quality and 30 as “satisfactory” quality (Supplementary Table 2). Of the two cohort studies, one was rated “good” quality and one “poor” quality (Supplementary Table 3). The single case-control study was rated as “good” quality (Supplementary Table 4).

### Publication bias

The publication bias for the prevalence of celiac disease in patients with TS was evaluated. The Doi plot is presented in Supplementary Figure 8. The LFK index for celiac disease in TS patients was −3.4, indicating major asymmetry. The funnel plot for this analysis is presented in Supplementary Figure 9. The Egger test result (*p* = 0.0125) indicated significant asymmetry. Using the trim-and-fill method, studies potentially omitted due to publication bias were added. For celiac disease in TS patients, 13 additional studies were imputed, leading to a revised prevalence estimate of 7.97% (95% CI: 6.44–9.84).

## Discussion

We conducted a systematic review and meta-analysis to examine the association between sex chromosome abnormalities and various AIDs. Through multiple studies, we explored correlations with specific conditions, identifying significant findings for certain AIDs. Previous meta-analyses have established significant associations between TS and conditions such as celiac disease [13] and Crohn’s disease [14]. Our study builds on these findings by examining a broader range of AIDs, offering a comprehensive overview of their prevalence in TS patients.

We observed notable differences in the prevalence of autoimmune conditions such as AIT, particularly HT, T1DM, Crohn’s disease, celiac disease, and alopecia areata. Given the frequent comorbidity among AIDs [74], comprehensive management is crucial, requiring regular monitoring and follow-up to detect and address complications early. Interestingly, research suggests a link between some of these conditions (HT, T1DM, and alopecia areata) and the X chromosome, providing insights into possible genetic underpinnings [75–77]. However, conditions like vitiligo and psoriasis showed comparatively smaller differences in prevalence or severity. We conducted an in-depth analysis of specific AIDs, classifying them by type to further elucidate their relationships.

### Thyroid diseases (autoimmune thyroiditis, Hashimoto’s thyroiditis, and Graves’ disease)

AIT is a chronic disease marked by persistent inflammation of the thyroid gland due to an autoimmune reaction against thyroid antigens. This condition can lead to the destruction of thyroid cells and hypothyroidism in approximately 30% of cases [78]. The pooled prevalence of AIT across included studies was 21.61% (95% CI: 12.85–30.37, I^2^ = 88%, *p* < 0.00001), which is significantly higher than the 2–5% prevalence observed in the general population [79]. In addition, we investigated HT and GD as representative forms of hypothyroidism and hyperthyroidism, respectively, in the context of AIT [80]. The pooled prevalence of HT derived from included studies was 25.78% (95% CI: 14.29–37.28, I^2^ = 88%, *p* < 0.0001), also notably higher than the 2% observed in the general population [81]. The pooled prevalence of GD, however, was 2.08% (95% CI: 1.03–3.14, I^2^ = 0%, *p* < 0.0001), closely aligning with the general population rate of 1.5% [82].

In one study, it was found that 20% of TS patients had subclinical hypothyroidism, while 70.4% exhibited antithyroid antibodies [83]. Additionally, anti-NIS antibodies were detected in some patients and were significantly associated with hypothyroidism (*p* < 0.04). However, since these antibodies did not inhibit iodine uptake, the authors proposed that anti-NIS antibodies may serve more as markers of early autoimmune activity rather than directly causing thyroid dysfunction. The underlying cause of increased risk of autoimmune diseases in Turner syndrome remains unclear. A review exploring the reasons behind the higher prevalence of autoimmune thyroid diseases in women points to the influence of not only genetic factors linked to the sex chromosomes but also hormonal and environmental factors [84]. Certain genes located on the X chromosome are known to be crucial for immune regulation. In many TS patients, autoimmune thyroiditis can initially be asymptomatic but may eventually progress to overt thyroid dysfunction [31], The International TS Study Group Consensus guidelines (2016) recommend screening for hypothyroidism at the time of diagnosis and continuing annually with (free) T4 and TSH measurements starting from early childhood and throughout the lifespan [85] Therefore, as these guidelines emphasize, annual thyroid function screening is essential in TS patients.

### Type 1 diabetes mellitus

Diabetes Mellitus (DM) is one of the most prevalent diseases worldwide, affecting 43 per 10,000 people [86]. T1DM, a form of DM, is classified as an AID, in which pancreatic beta cells are targeted by autoantibodies. The pooled prevalence of T1DM across included studies was 1.32% (95% CI: 0.64–2.00, I^2^ = 0%, *p* < 0.001), a significantly higher rate compared to the 0.42% prevalence observed in the general population [86].

Type 1 DM is a type of organ-specific autoimmune disease that involves the targeted destruction of pancreatic β-cells [87]. Epidemiological data suggest that patients with TS face up to a tenfold higher risk of developing type 1 DM at any age compared to the general population [85]. Even in the absence of overt diabetes, TS patients often exhibit various disruptions in glucose metabolism, such as hyperinsulinemia, insulin resistance, decreased insulin secretion, and impaired glucose tolerance [88,89] These disturbances likely reflect a combination of impaired early-phase insulin release and reduced β-cell responsiveness. The 2016 International TS Study Group Consensus guidelines advise lifelong annual screening for HbA1c, with or without fasting plasma glucose, beginning at age 10 years [85]. Moreover, the group recommends that if diabetes is diagnosed, testing for type 1 DM–related autoantibodies should be conducted to establish the autoimmune nature of the disease. Hamza et al. (2013) advocated for GAD-65 antibody testing in all TS patients with newly diagnosed diabetes to confirm the autoimmune basis [39]. These screening recommendations underscore the importance of early identification and proactive management of T1DM in this particularly high-risk population.

### Crohn’s disease

Crohn’s disease is a type of inflammatory bowel disease characterized by chronic inflammation of any part of the gastrointestinal tract, most commonly the ileum and colon [90,91]. The pooled prevalence of Crohn’s disease from the included studies was 1.13% (95% CI: 0.92–1.34, I^2^ = 0%, *p* < 0.00001), significantly higher than the 0.1%−0.3% prevalence reported for the general populations of North America and Europe [92]. Despite analysing data from various global regions, the prevalence of Crohn’s disease remained consistently elevated among TS patients, reflecting the recognized higher rates in North America and Europe. In one study from Japan, the prevalence of Crohn’s disease among TS patients was 0.8%, exceeding the rates observed in general populations of North America and Europe [36]. These findings suggest a potential correlation between TS and an increased incidence of Crohn’s disease. Crohn’s disease appears to be more common than ulcerative colitis in this population. It also tends to present at a younger age and with more severe symptoms compared to the general population [1]. Therefore, it is advised that any patient presenting with abdominal pain, unexplained weight loss, diarrhoea, and/or intestinal bleeding should be evaluated for possible inflammatory bowel disease [1].

### Celiac disease

Celiac disease, also known as ‘celiac sprue,’ is a chronic inflammatory disorder of the small intestine triggered by the ingestion of dietary gluten [93]. The pooled prevalence of celiac disease across included studies was 5.89% (95% CI: 4.49–7.28, I^2^ = 85%, *p* < 0.001), which is significantly higher than the 1% prevalence observed in the general population [94].

A study based on biopsy data reported that the relative risk of celiac disease is twofold higher in children under 5 years of age and fivefold higher in those over 10 years, suggesting an age-related increase in prevalence among TS patients [1]. Similar to autoimmune thyroid disease and type 1 DM, the heightened susceptibility to celiac disease in TS patients is thought to result from X chromosome haploinsufficiency, maternal origin of the X chromosome, increased production of pro-inflammatory cytokines (such as IL-6), and reduced anti-inflammatory cytokines (including IL-10 and TGF-β) [2]. Celiac disease is strongly linked to the HLA-DQ2 and HLA-DQ8 genes [3]. HLA typing has been proposed as a useful first-line screening method in high-risk populations like TS patients [4]. If both HLA-DQ2 and HLA-DQ8 are negative, the likelihood of developing celiac disease in the future is very low, and HLA testing can also help clarify uncertain diagnoses [5]. However, these HLA genes account for only around 40% of the genetic susceptibility, suggesting that other factors such as single-nucleotide polymorphisms (SNPs) and epigenetic modifications may also contribute [6]. The International TS Study Group Consensus guidelines recommend initiating celiac disease screening with transglutaminase antibody testing at ages 2–3 years, to be repeated every two years throughout childhood and as needed based on adult symptoms [4]. Early and consistent screening, as these guidelines suggest, is essential to reduce complications and improve long-term health outcomes in TS patients with celiac disease. Given the potential for associated complications, timely follow-up for TS patients is crucial, and a gluten-free diet should be recommended to manage symptoms and prevent further complications [93].

### Alopecia areata, vitiligo, and psoriasis

The pooled prevalence of alopecia areata across included studies was 0.84% (95% CI: −0.02 − 1.70, I^2^ = 58%, *p* = 0.05), compared to a global prevalence of approximately 0.1-0.2% [95]. This suggests that TS patients are more likely to develop Alopecia Areata than the general population. The pooled prevalence of vitiligo from included studies was 0.84% (95% CI: 0.24–1.44, I^2^ = 23%, *p* < 0.006), a rate significantly lower than that of the general population (2%). The pooled prevalence of psoriasis from included studies was 1.14% (95% CI: 0.18–2.11, I^2^ = 100%, *p* < 0.001), showing no significant difference from the general population prevalence, which ranges from 0.14% to 3.6% [96].

The association between TS and T cell–mediated autoimmune diseases, including psoriasis, alopecia areata, and vitiligo, has been documented [62]. This increased susceptibility is thought to result from haploinsufficiency of X chromosome–linked genes, leading to downstream immune effects such as increased CD8+ T cell activity and reduced regulatory T cell populations [62]. Interestingly, the lower prevalence of vitiligo in TS patients is counterintuitive and suggests that immune dysregulation in TS does not uniformly increase the risk for all autoimmune diseases. This unexpected finding indicates that certain autoimmune pathways may be suppressed or differently regulated in TS, highlighting the need for further research into disease-specific immunogenetic mechanisms.

## Limitations

Most of the included studies are cross-sectional, limiting the ability to infer causal relationships between TS and AIDs. Longitudinal studies are needed to better understand the progression and development of AIDs in this population over time. While this study initially aimed to assess a range of sex chromosome abnormalities, the limited data on conditions beyond TS led to a primary focus on TS, thereby limiting the generalizability of the findings to other abnormalities, such as KS or other X/Y chromosomal variations. Additionally, the availability of studies on specific AIDs varies. For conditions with limited data, such as alopecia areata and vitiligo, the prevalence estimates may not fully represent the TS population. Reliance on published literature may have introduced publication bias, as studies with significant findings are more likely to be published than those with null results. Furthermore, the prevalence of AIDs can be influenced by confounding factors such as ethnicity, age, and environmental exposure, which were not consistently adjusted for across the included studies.

## Conclusion

This study presents a comprehensive meta-analysis of autoimmune condition prevalence in TS, representing a novel contribution in an area previously lacking such analysis. Our findings reveal a complex profile of autoimmune risk in TS patients. Prevalence rates were significantly higher for several AIDs compared to the general population, particularly for AIT (especially HT), T1DM, Crohn’s disease, and celiac disease. In contrast, the prevalence of vitiligo was significantly lower, while the rates for alopecia areata and psoriasis were not significantly different. These findings contribute valuable insights to the field and underscore the importance of personalized healthcare approaches in managing autoimmune conditions among patients with sex chromosome abnormalities. Sustained research efforts and interdisciplinary collaboration are essential to improving health outcomes for TS patients with autoimmune conditions.

## Supplementary Material

Supplementary Materials.docx

PRISMA checklist.docx

## Data Availability

No new data were generated or analyzed in this study. All data are derived from previously published studies.
