# Supplementary material for: Global prevalence of autoimmune diseases in turner syndrome: a systematic review and meta-analysis
Source: Ann Med. 2025 Nov 16;57(1):2573143. doi: 10.1080/07853890.2025.2573143 (PMC12624938; doi:10.1080/07853890.2025.2573143)

**Supplementary Table 1.** Final search strategy for each database

| **Database** | **Search strategy** |
| --- | --- |
| Web of Science | (TI=(prevalence) OR AB=(prevalence) OR TI=(incidence) OR AB=(incidence))  AND  (TI=(X chromosome) OR AB=(X chromosome) OR TI=(chromosome X) OR AB=(chromosome X) OR TI=(sex chromosome*) OR AB=(sex chromosome*) OR TI=(Turner syndrome) OR AB=(Turner syndrome) OR TI=(monosomy X) OR AB=(monosomy X) OR TI=(Triple X syndrome) OR AB=(Triple X syndrome) OR TI=(XXX) OR AB=(XXX) OR TI=(Tetrasomy X) OR AB=(Tetrasomy X) OR TI=(XXXX) OR AB=(XXXX) OR TI=(Pentasomy X) OR AB=(Pentasomy X) OR TI=(XXXXX) OR AB=(XXXXX) OR TI=(Klinefelter syndrome) OR AB=(Klinefelter syndrome) OR TI=(XXY) OR AB=(XXY) OR TI=(XXXY) OR AB=(XXXY) OR TI=(XXXXY) OR AB=(XXXXY))  AND  (TI=(autoimmune) OR AB=(autoimmune) OR TI=(atopic dermatitis) OR AB=(atopic dermatitis) OR TI=(eczema) OR AB=(eczema) OR TI=(vitiligo) OR AB=(vitiligo) OR TI=(psoriasis) OR AB=(psoriasis) OR TI=(Rheumatoid arthritis) OR AB=(Rheumatoid arthritis) OR TI=(diabetes) OR AB=(diabetes) OR TI=(inflammatory bowel disease) OR AB=(inflammatory bowel disease) OR TI=(Crohn's disease) OR AB=(Crohn's disease) OR TI=(ulcerative colitis) OR AB=(ulcerative colitis) OR TI=(behcet) OR AB=(behcet) OR TI=(lupus) OR AB=(lupus) OR TI=(SLE) OR AB=(SLE) OR TI=(sjogren's syndrome) OR AB=(sjogren's syndrome) OR TI=(myasthenia gravis) OR AB=(myasthenia gravis) OR TI=(thyroid) OR AB=(thyroid) OR TI=(Hashimoto's disease) OR AB=(Hashimoto's disease) OR TI=(Hashimoto's thyroiditis) OR AB=(Hashimoto's thyroiditis) OR TI=(hyperthyroidism) OR AB=(hyperthyroidism) OR TI=(hypothyroidism) OR AB=(hypothyroidism) OR TI=(Graves' disease) OR AB=(Graves' disease) OR TI=(fibromyalgia) OR AB=(fibromyalgia) OR TI=(multiple sclerosis) OR AB=(multiple sclerosis) OR TI=(alopecia) OR AB=(alopecia) OR TI=(collagen disease) OR AB=(collagen disease) OR TI=(Kikuchi's disease) OR AB=(Kikuchi's disease) OR TI=(Guillain–Barre syndrome) OR AB=(Guillain–Barre syndrome) OR TI=(vasculitis) OR AB=(vasculitis) OR TI=(Polyarteritis Nodosa) OR AB=(Polyarteritis Nodosa) OR TI=(Scleroderma) OR AB=(Scleroderma)) |
| Scopus | TITLE-ABS-KEY(prevalence) OR TITLE-ABS-KEY(incidence)  AND  (TITLE-ABS-KEY(X chromosome) OR TITLE-ABS-KEY(chromosome X) OR TITLE-ABS-KEY(sex chromosome*) OR TITLE-ABS-KEY(Turner syndrome) OR TITLE-ABS-KEY(monosomy X) OR TITLE-ABS-KEY(Triple X syndrome) OR TITLE-ABS-KEY(XXX) OR TITLE-ABS-KEY(Tetrasomy X) OR TITLE-ABS-KEY(XXXX) OR TITLE-ABS-KEY(Pentasomy X) OR TITLE-ABS-KEY(XXXXX) OR TITLE-ABS-KEY(Klinefelter syndrome) OR TITLE-ABS-KEY(XXY) OR TITLE-ABS-KEY(XXXY) OR TITLE-ABS-KEY(XXXXY))  AND  (TITLE-ABS-KEY(autoimmune) OR TITLE-ABS-KEY(atopic dermatitis) OR TITLE-ABS-KEY(eczema) OR TITLE-ABS-KEY(vitiligo) OR TITLE-ABS-KEY(psoriasis) OR TITLE-ABS-KEY(Rheumatoid arthritis) OR TITLE-ABS-KEY(diabetes) OR TITLE-ABS-KEY(inflammatory bowel disease) OR TITLE-ABS-KEY(Crohn's disease) OR TITLE-ABS-KEY(ulcerative colitis) OR TITLE-ABS-KEY(behcet) OR TITLE-ABS-KEY(lupus) OR TITLE-ABS-KEY(SLE) OR TITLE-ABS-KEY(sjogren's syndrome) OR TITLE-ABS-KEY(myasthenia gravis) OR TITLE-ABS-KEY(Hashimoto's disease) OR TITLE-ABS-KEY(Hashimoto's thyroiditis) OR TITLE-ABS-KEY(hyperthyroidism) OR TITLE-ABS-KEY(hypothyroidism) OR TITLE-ABS-KEY(Graves' disease) OR TITLE-ABS-KEY(fibromyalgia) OR TITLE-ABS-KEY(multiple sclerosis) OR TITLE-ABS-KEY(alopecia) OR TITLE-ABS-KEY(collagen disease) OR TITLE-ABS-KEY(Kikuchi's disease) OR TITLE-ABS-KEY(Guillain–Barre syndrome) OR TITLE-ABS-KEY(vasculitis) OR TITLE-ABS-KEY(Polyarteritis Nodosa) OR TITLE-ABS-KEY(Scleroderma)) |
| MEDLINE - Embase | ('x chromosome':ab,ti OR 'chromosome x':ab,ti OR 'sex chromosome*':ab,ti OR 'turner syndrome':ab,ti OR 'monosomy x':ab,ti OR 'triple x syndrome':ab,ti OR xxx:ab,ti OR 'tetrasomy x':ab,ti OR xxxx:ab,ti OR 'pentasomy x':ab,ti OR xxxxx:ab,ti OR 'klinefelter syndrome':ab,ti OR xxy:ab,ti OR xxxy:ab,ti OR xxxxy:ab,ti)  AND  (prevalence:ab,ti OR incidence:ab,ti)  AND  ((autoimmune:ab,ti OR 'atopic dermatitis':ab,ti OR eczema:ab,ti OR vitiligo:ab,ti OR psoriasis:ab,ti OR 'rheumatoid arthritis':ab,ti OR diabetes:ab,ti OR 'inflammatory bowel disease':ab,ti OR 'crohns disease':ab,ti OR 'ulcerative colitis':ab,ti OR behcet:ab,ti OR lupus:ab,ti OR sle:ab,ti OR 'sjogrens syndrome':ab,ti OR 'myasthenia gravis':ab,ti OR 'hashimotos disease':ab,ti OR 'hashimotos thyroiditis':ab,ti OR hyperthyroidism:ab,ti OR hypothyroidism:ab,ti OR 'graves disease':ab,ti OR fibromyalgia:ab,ti OR 'multiple sclerosis':ab,ti OR alopecia:ab,ti OR 'collagen disease':ab,ti OR 'kikuchis disease':ab,ti OR 'guillain-barre syndrome':ab,ti OR vasculitis:ab,ti OR 'polyarteritis nodosa':ab,ti OR scleroderma:ab,ti) OR thyroid:ab,ti)  AND  ([article]/lim OR [article in press]/lim)  AND  [humans]/lim |

* The Embase database was utilized for the search as it encompasses the search conducted in MEDLINE.

**Supplementary Table 2.** Risk of bias assessment for cross-sectional studies – Newcastle–Ottawa Scale

| **Source** | **Selection** | | | | **Comparability based on design and analysis** | **Outcome** | | **Total** | **Assessment** |
| --- | --- | --- | --- | --- | --- | --- | --- | --- | --- |
|  | **Representativeness of the sample** | **Sample size** | **Non-respondents** | **Ascertainment of the exposure** |  | **Assessment of outcome** | **Statistical test** |  |  |
| Naessén 2024 | ★ |  |  | ★★ | ★★ | ★ | ★ | **7** | Good |
| Witkowska-Krawczak 2023 |  |  |  | ★★ | ★★ | ★ | ★ | **6** | Satisfactory |
| Said 2022 |  |  |  | ★★ | ★★ | ★ | ★ | **6** | Satisfactory |
| Abdel-Badie Salem 2021 |  |  |  | ★★ | ★★ | ★ | ★ | **6** | Satisfactory |
| Farquhar 2020 | ★ |  |  | ★★ | ★★ | ★ | ★ | **7** | Good |
| Yeşilkaya 2015 |  |  |  | ★★ | ★★ | ★ | ★ | **6** | Satisfactory |
| Grossi 2013 |  |  |  | ★★ | ★★ | ★ | ★ | **6** | Satisfactory |
| Wegiel 2019 |  |  |  | ★★ | ★★ | ★ | ★ | **6** | Satisfactory |
| Hanew 2018 | ★ |  |  | ★★ | ★★ | ★ | ★ | **7** | Good |
| Larizza 2016 |  |  |  | ★★ | ★★ | ★ | ★ | **6** | Satisfactory |
| Valenzise 2014 |  |  |  | ★★ | ★★ | ★ | ★ | **6** | Satisfactory |
| Hamza 2013 |  |  |  | ★★ | ★★ | ★ | ★ | **6** | Satisfactory |
| Bakalov 2012 |  |  |  | ★★ | ★★ | ★ | ★ | **6** | Satisfactory |
| Fukada 2009 |  |  |  | ★★ | ★★ | ★ | ★ | **6** | Satisfactory |
| Gravholt 1998 | ★ |  |  | ★★ | ★★ | ★ | ★ | **7** | Good |
| Lee 2023 | ★ | ★ |  | ★★ | ★★ | ★ | ★ | **8** | Good |
| Bonamico M. |  |  |  | ★★ | ★★ | ★ | ★ | **6** | Satisfactory |
| Ivarsson 1999 |  |  |  | ★★ | ★★ | ★ | ★ | **6** | Satisfactory |
| Gillett 2000 |  |  |  | ★★ | ★★ | ★ | ★ | **6** | Satisfactory |
| Rujner 2001 |  |  |  | ★★ | ★★ | ★ | ★ | **6** | Satisfactory |
| Bonamico M. | ★ |  |  | ★★ | ★★ | ★ | ★ | **7** | Good |
| Sakly 2005 |  |  |  | ★★ | ★★ | ★ | ★ | **6** | Satisfactory |
| Bahremand 2005 |  |  |  | ★★ | ★★ | ★ | ★ | **6** | Satisfactory |
| Bettendorf 2002 | ★ |  |  | ★★ | ★★ | ★ | ★ | **7** | Good |
| Ságodi 2006 |  |  |  | ★★ | ★★ | ★ | ★ | **6** | Satisfactory |
| Mortensen 2009 |  |  |  | ★★ | ★★ | ★ | ★ | **6** | Satisfactory |
| Frost 2009 |  |  |  | ★★ | ★★ | ★ | ★ | **6** | Satisfactory |
| Dias Mdo 2010 |  |  |  | ★★ | ★★ | ★ | ★ | **6** | Satisfactory |
| Nabhan 2011 |  |  |  | ★★ | ★★ | ★ | ★ | **6** | Satisfactory |
| Freriks 2011 |  |  |  | ★★ | ★★ | ★ | ★ | **6** | Satisfactory |
| Nadeem 2013 |  |  |  | ★★ | ★★ | ★ | ★ | **6** | Satisfactory |
| Goldacre 2014 |  |  |  | ★★ | ★★ | ★ | ★ | **6** | Satisfactory |
| Rutigliano 2015 |  |  |  | ★★ | ★★ | ★ | ★ | **6** | Satisfactory |
| Hirschfield 2008 | ★ | ★ |  | ★★ | ★★ | ★ | ★ | **8** | Good |
| Baz Ouidad 2018 | ★ |  |  | ★★ | ★★ | ★ | ★ | **7** | Good |
| Kammoun 2012 |  |  |  | ★★ | ★★ | ★ | ★ | **6** | Satisfactory |
| Berglund 2019 | ★ |  |  | ★★ | ★★ | ★ | ★ | **7** | Good |
| Bessahraoui 2014 |  |  |  | ★★ | ★★ | ★ | ★ | **6** | Satisfactory |
| Dumitrescua 2018 |  |  |  | ★★ | ★★ | ★ | ★ | **6** | Satisfactory |
| Elechi 2018 |  |  |  | ★★ | ★★ | ★ | ★ | **6** | Satisfactory |
| Stenberg 2007 | ★ |  |  | ★★ | ★★ | ★ | ★ | **7** | Good |

*Very good quality: 9-10 points, Good quality: 7-8 points, Satisfactory quality: 5-6 points, Unstatisfactory quality: 0-4 points

**Supplementary Table 3.** Risk of bias assessment for case control studies – Newcastle–Ottawa Scale

| **Source** | **Selection** | | | | **Comparability based on design and analysis** | **Exposure** | | | **Total** | **Assessment** |
| --- | --- | --- | --- | --- | --- | --- | --- | --- | --- | --- |
|  | **Representativeness of the sample** | **Sample size** | **Non-respondents** | **Ascertainment of the exposure** |  | **Ascertainment of exposure** | **Same method of ascertainment for cases and controls** | **Non-Response rate** |  |  |
| Mårild 2016 | ★ | ★ | ★ | ★ | ★★ | ★ | ★ |  | 8 | Good |
| Gawlik 2018 | ★ | ★ |  | ★ | ★★ | ★ |  |  | 6 | Poor |

* Good quality: 3 or 4 points in selection domain AND 1 or 2 points in compatibility domain AND 2 or 3 points in outcome/exposure domain, Fair quality: 2 points in selection domain AND 1 or 2 points in comparability domain AND 2 or 3 points in outcome/exposure domain, Poor quality: 0 or 1 point in selection domain OR 0 points in comparability domain OR 0 or 1 point in outcome/exposure domain

**Supplementary Table 4.** Risk of bias assessment for cohort studies – Newcastle–Ottawa Scale

| **Source** | **Selection** | | | | **Comparability based on design and analysis** | **Outcome** | | | **Total** | **Assessment** |
| --- | --- | --- | --- | --- | --- | --- | --- | --- | --- | --- |
|  | **Representativeness of the exposed cohort** | **Selection of the non-exposed cohort** | **Ascertainment of exposure** | **Demonstration that outcome of interest was not present at start of study** |  | **Assessment of outcome** | **Was follow-up long enough for outcomes to occur** | **Adequacy of follow up of cohorts** |  |  |
| Stagi 2014 | ★ | ★ | ★ | ★ | ★★ |  | ★ | ★ | 8 | Good |

* Good quality: 3 or 4 points in selection domain AND 1 or 2 points in compatibility domain AND 2 or 3 points in outcome/exposure domain, Fair quality: 2 points in selection domain AND 1 or 2 points in comparability domain AND 2 or 3 points in outcome/exposure domain, Poor quality: 0 or 1 point in selection domain OR 0 points in comparability domain OR 0 or 1 point in outcome/exposure domain

Supplementary figure 1. Forest plot showing the prevalence of Hashimoto's thyroiditis in Turner syndrome


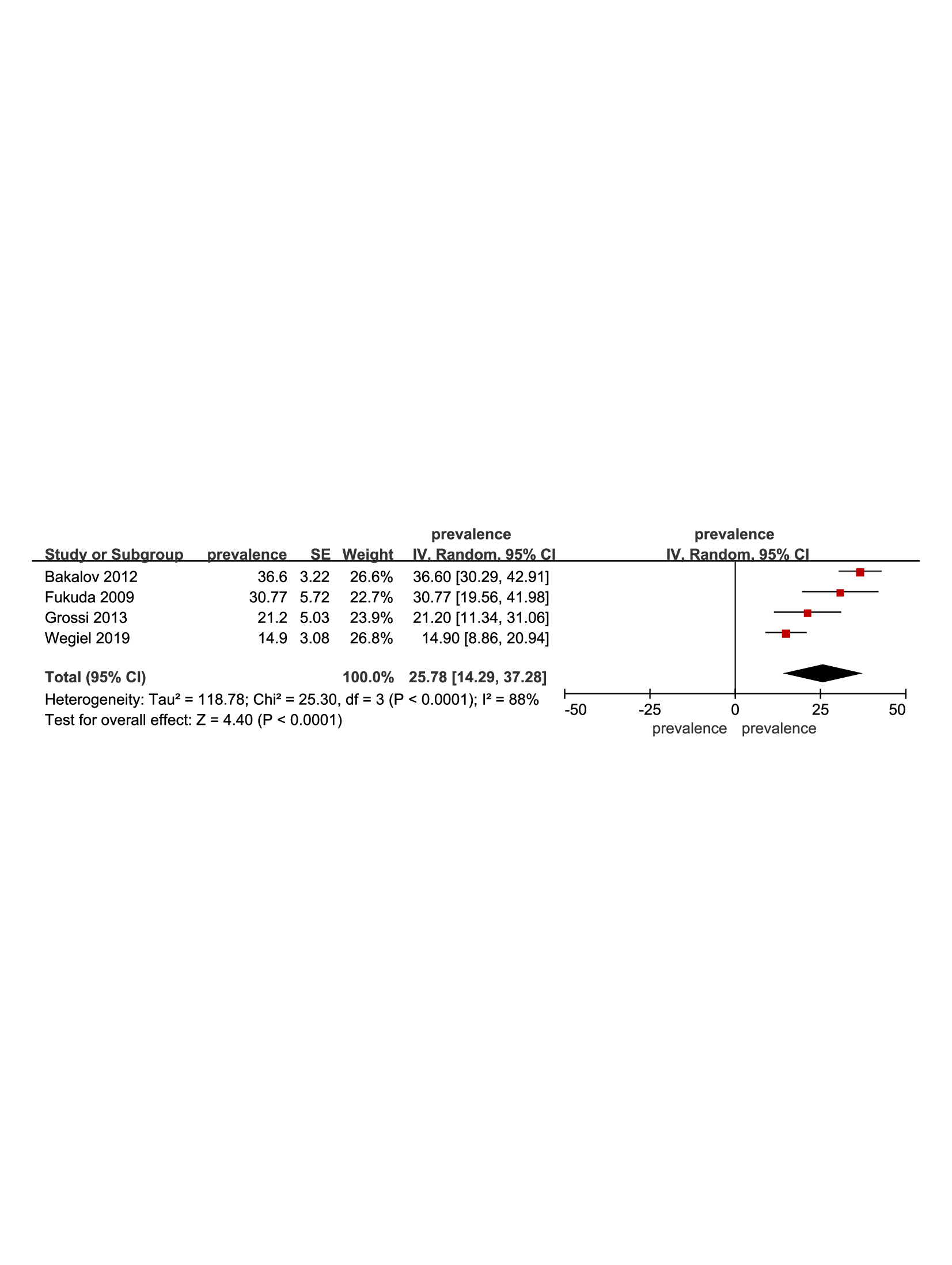


**Supplementary figure 2.** Forest plot showing the prevalence of Graves' disease in Turner syndrome


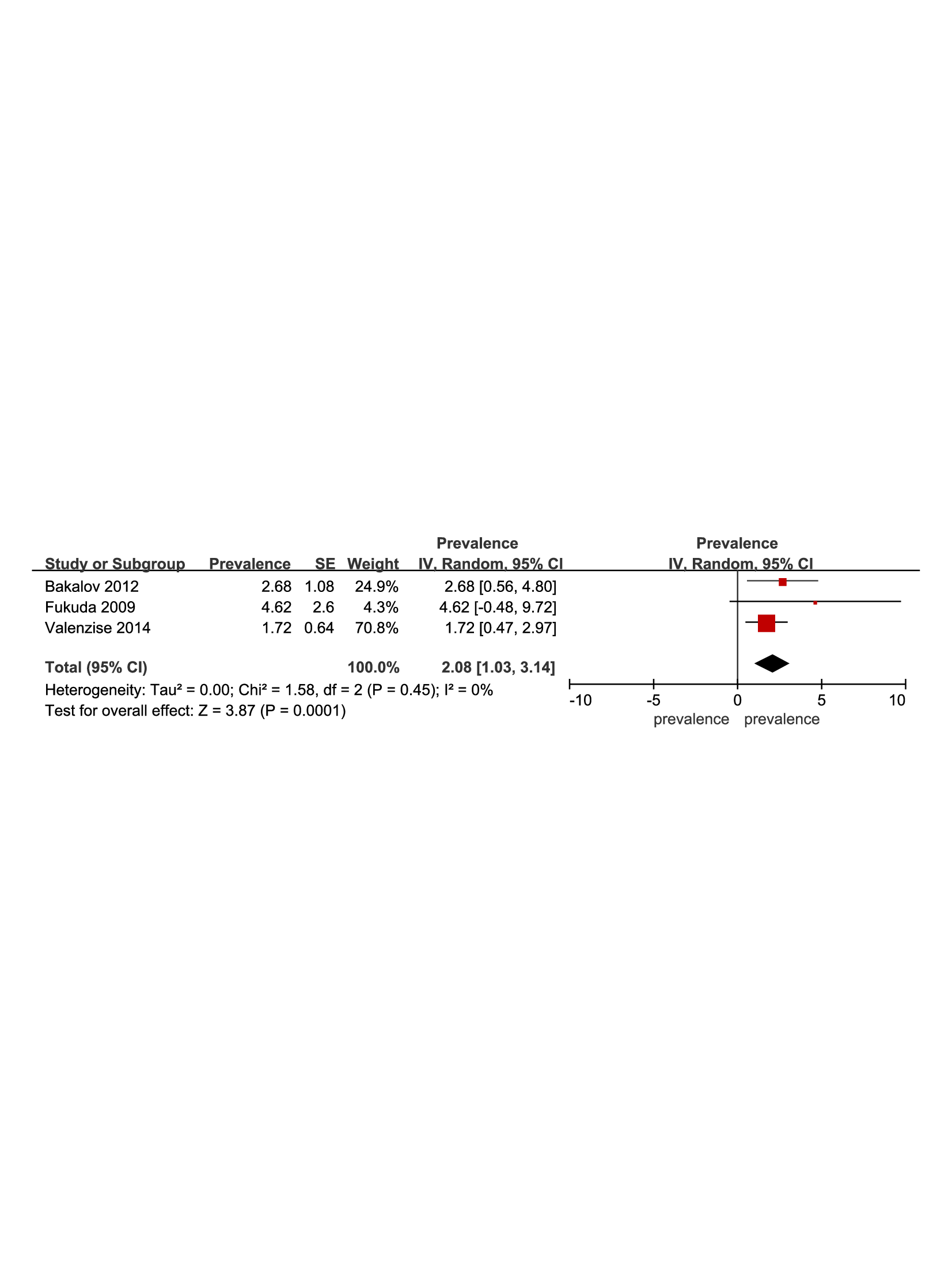


**Supplementary figure 3.** Forest plot showing the prevalence of type 1 diabetes mellitus in Turner syndrome


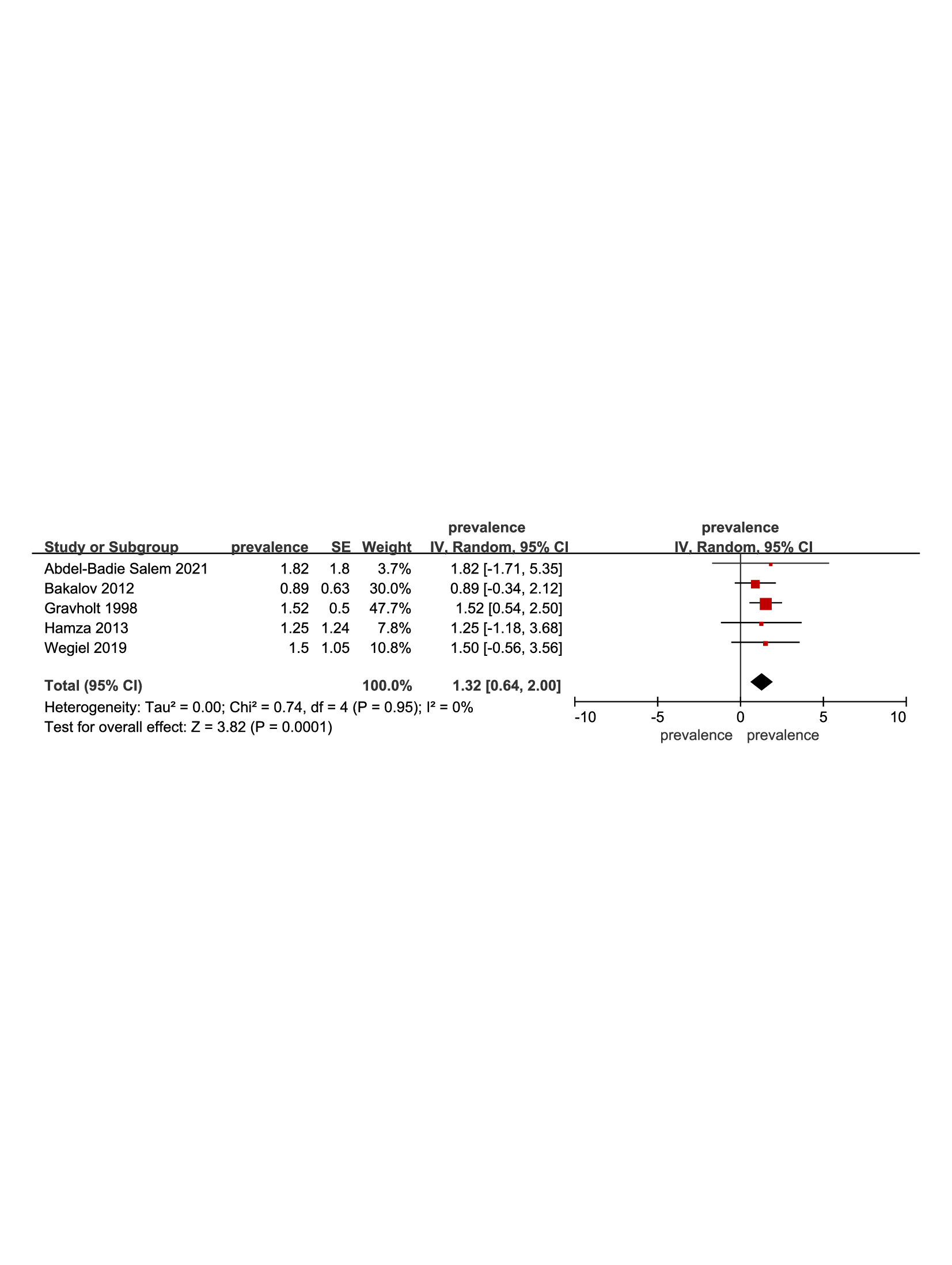


**Supplementary figure 4.** Forest plot showing the prevalence of Crohn’s disease in Turner syndrome


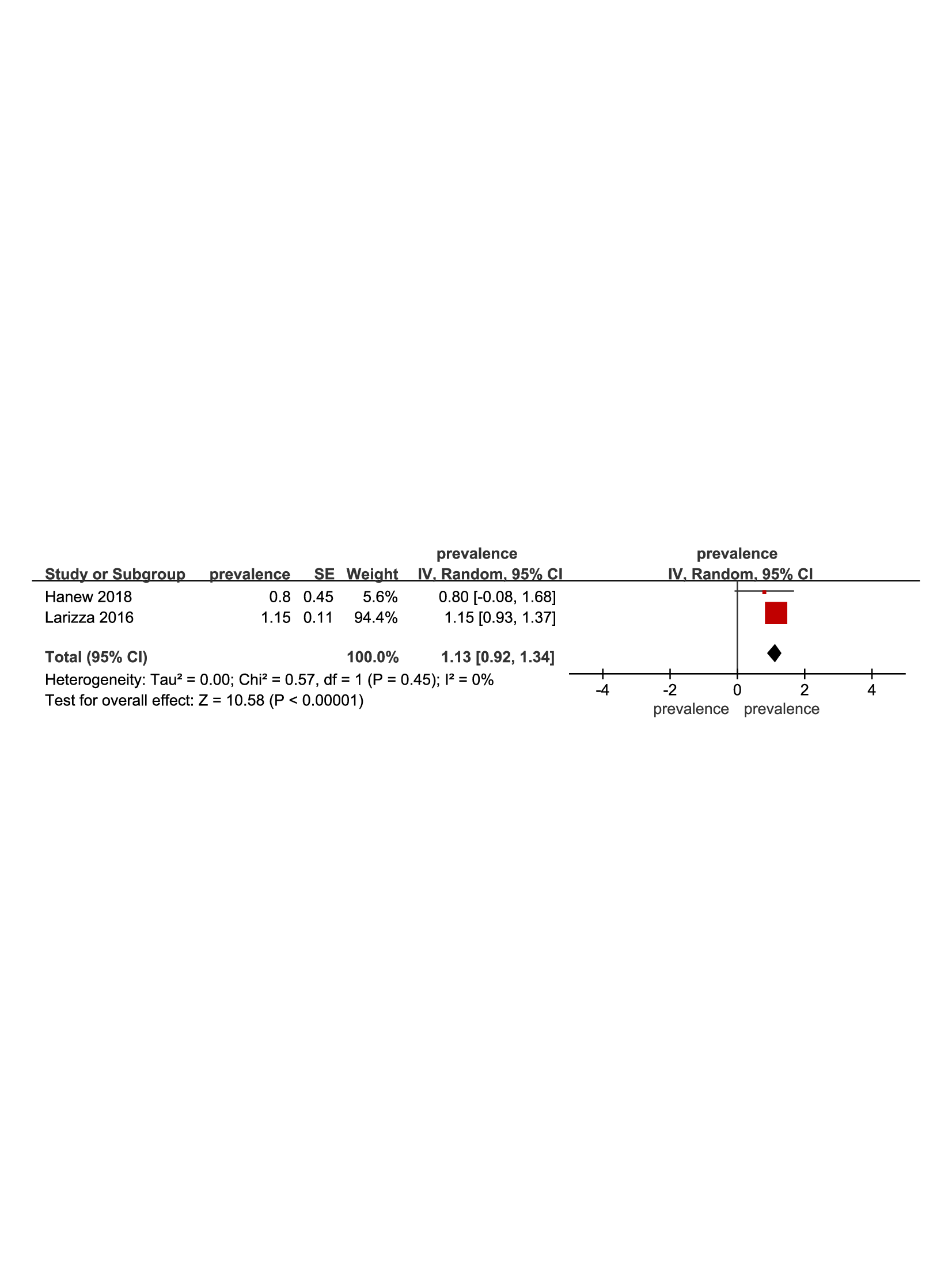


**Supplementary figure 5.** Forest plot showing the prevalence of alopecia areata in Turner syndrome


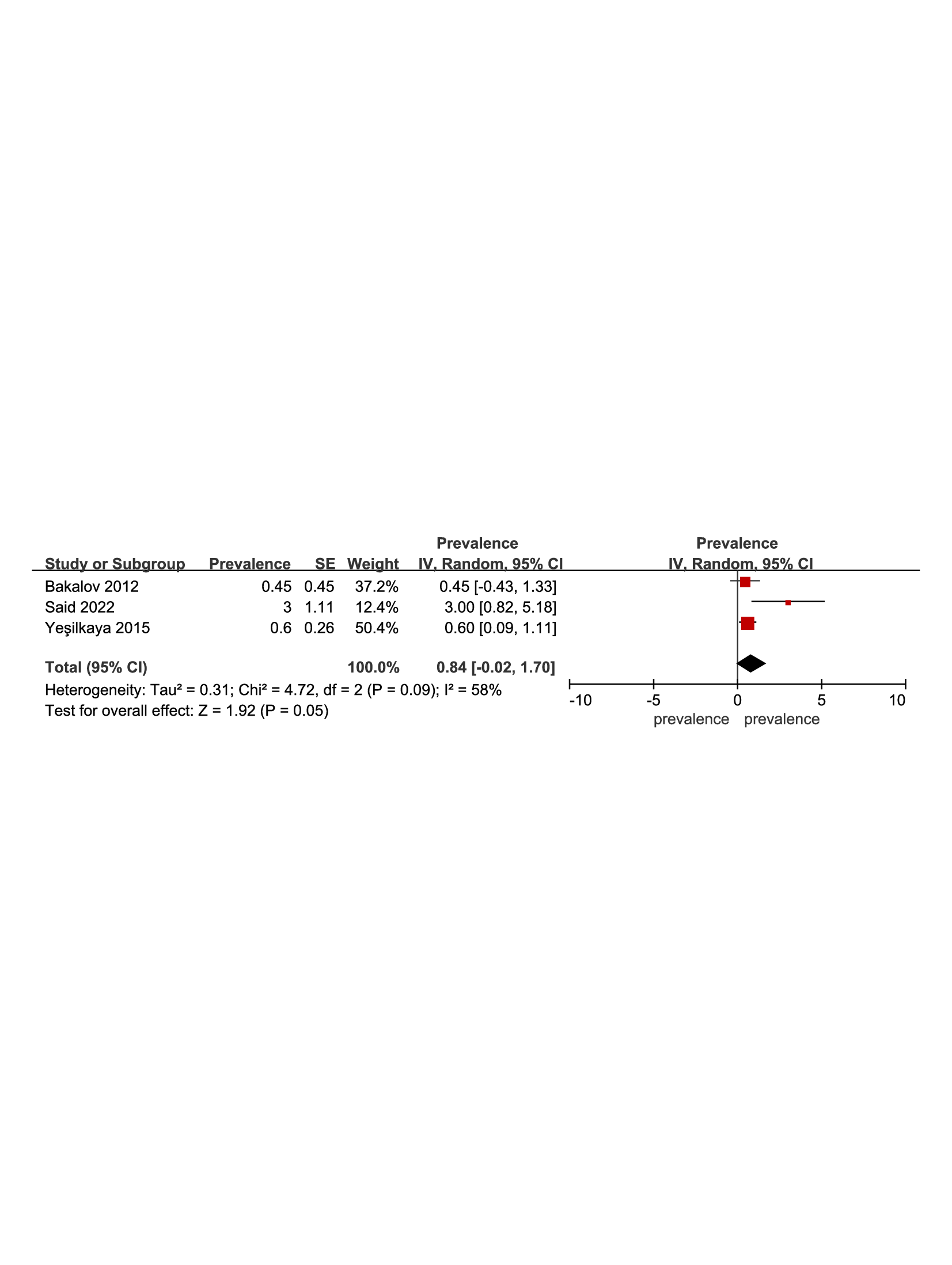


**Supplementary figure 6.** Forest plot showing the prevalence of vitiligo in Turner syndrome


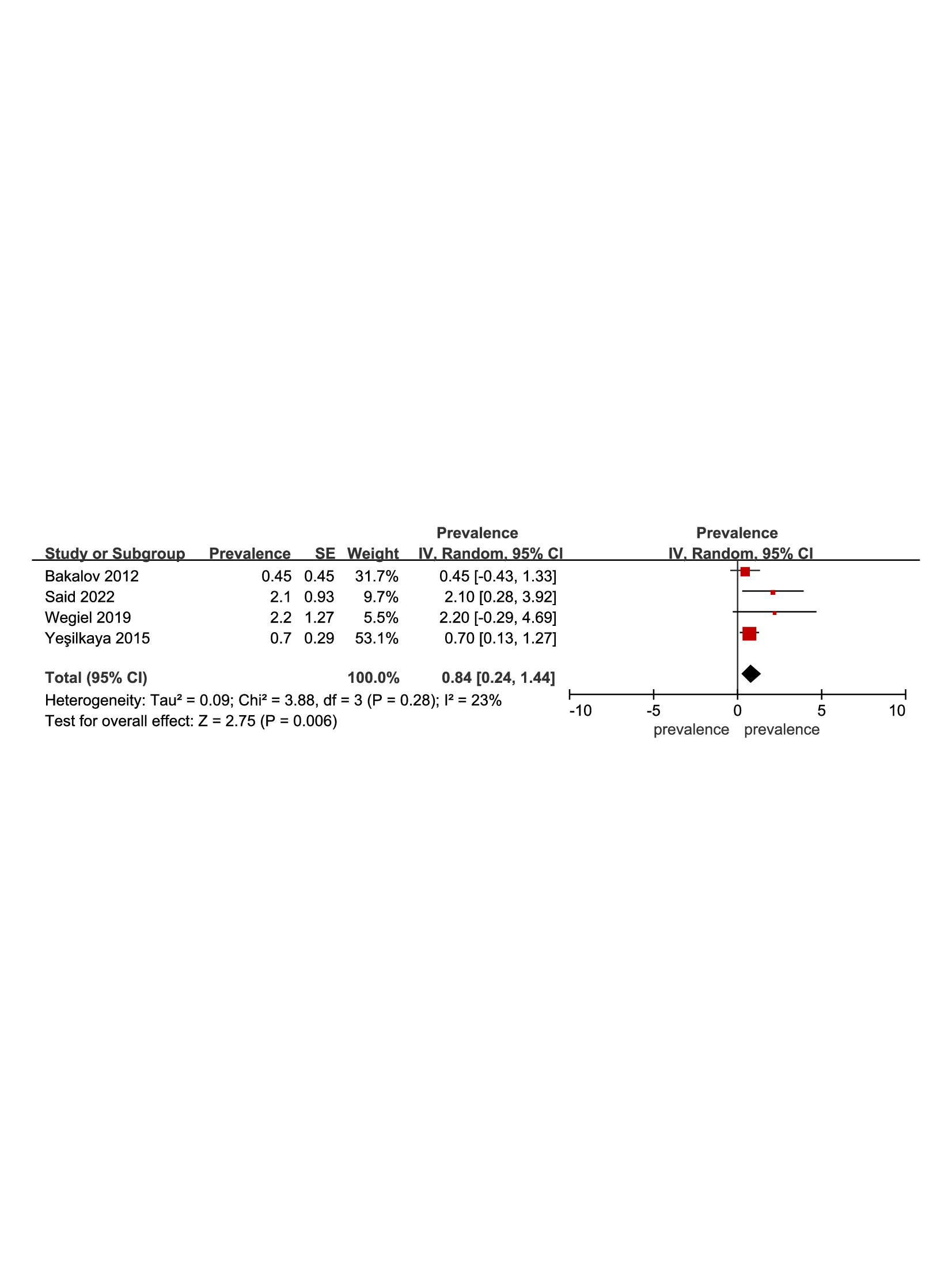


Supplementary figure 7. Forest plot showing the prevalence of psoriasis in Turner syndrome


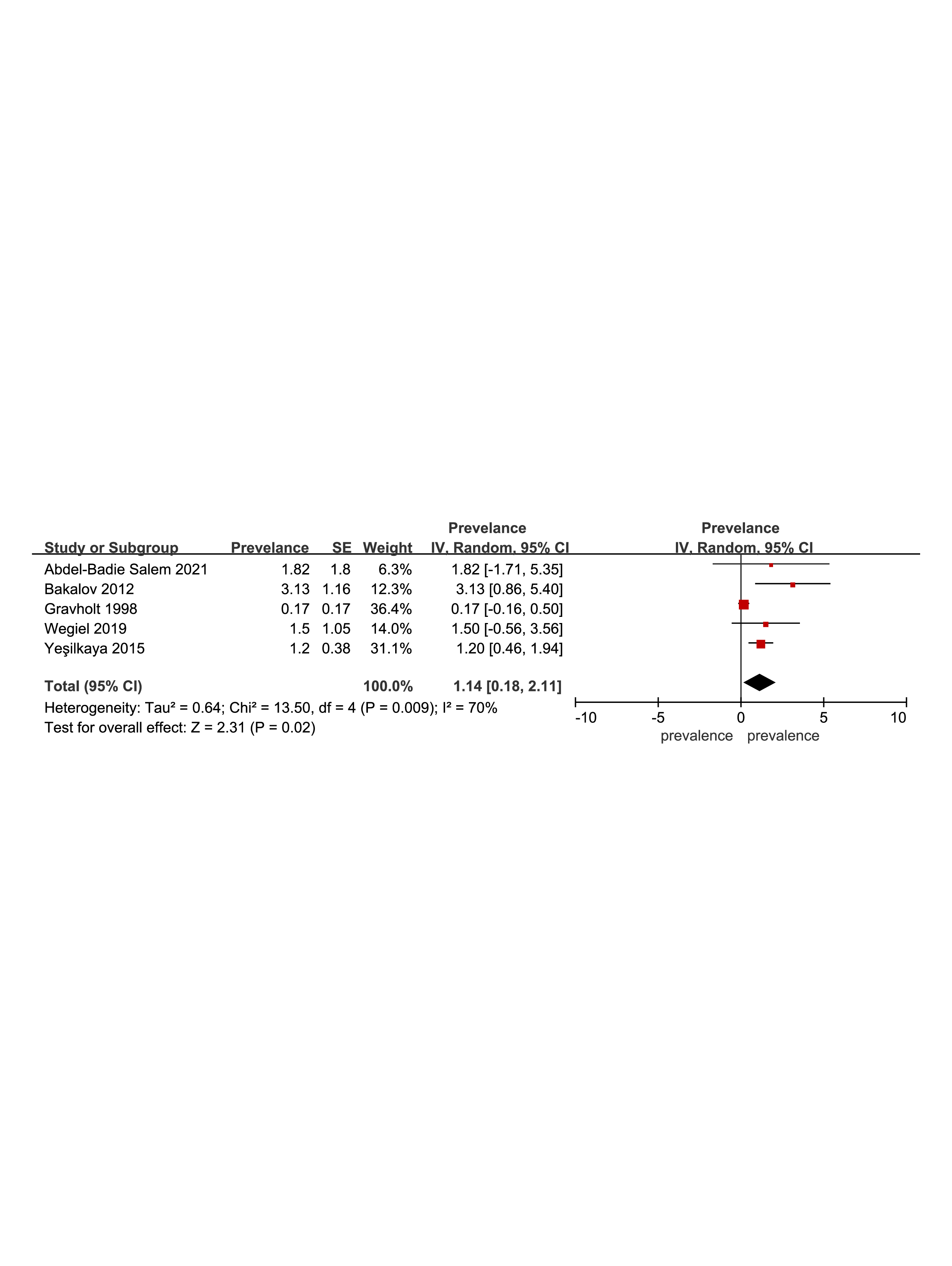


**Supplementary Figure 8.** The DOI plot and the Luis Furuya–Kanamori (LFK) index for assessing publication bias in studies on celiac disease in patients with Turner syndrome


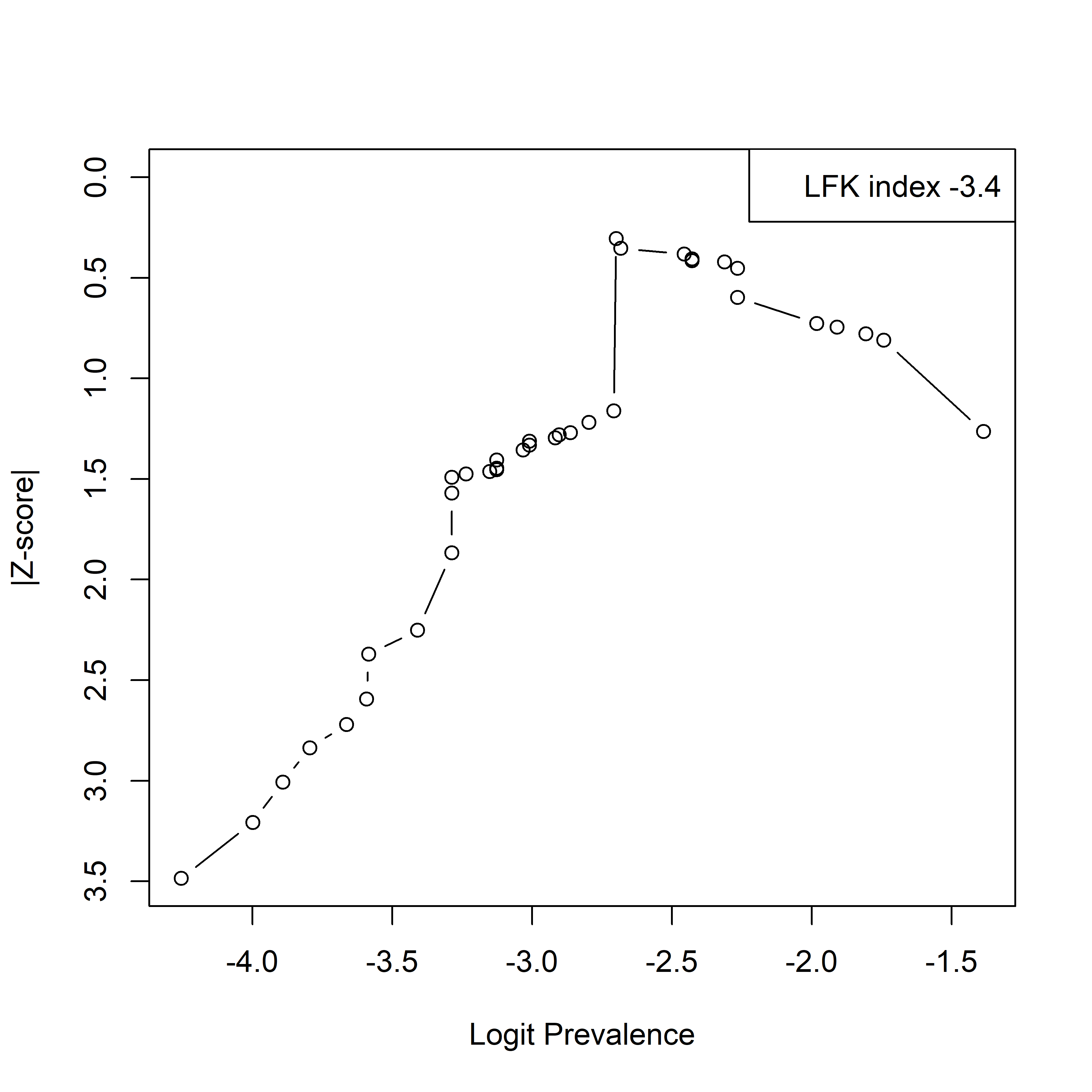


**Supplementary Figure 9.** Funnel plot for the prevalence of celiac disease in patients with Turner syndrome


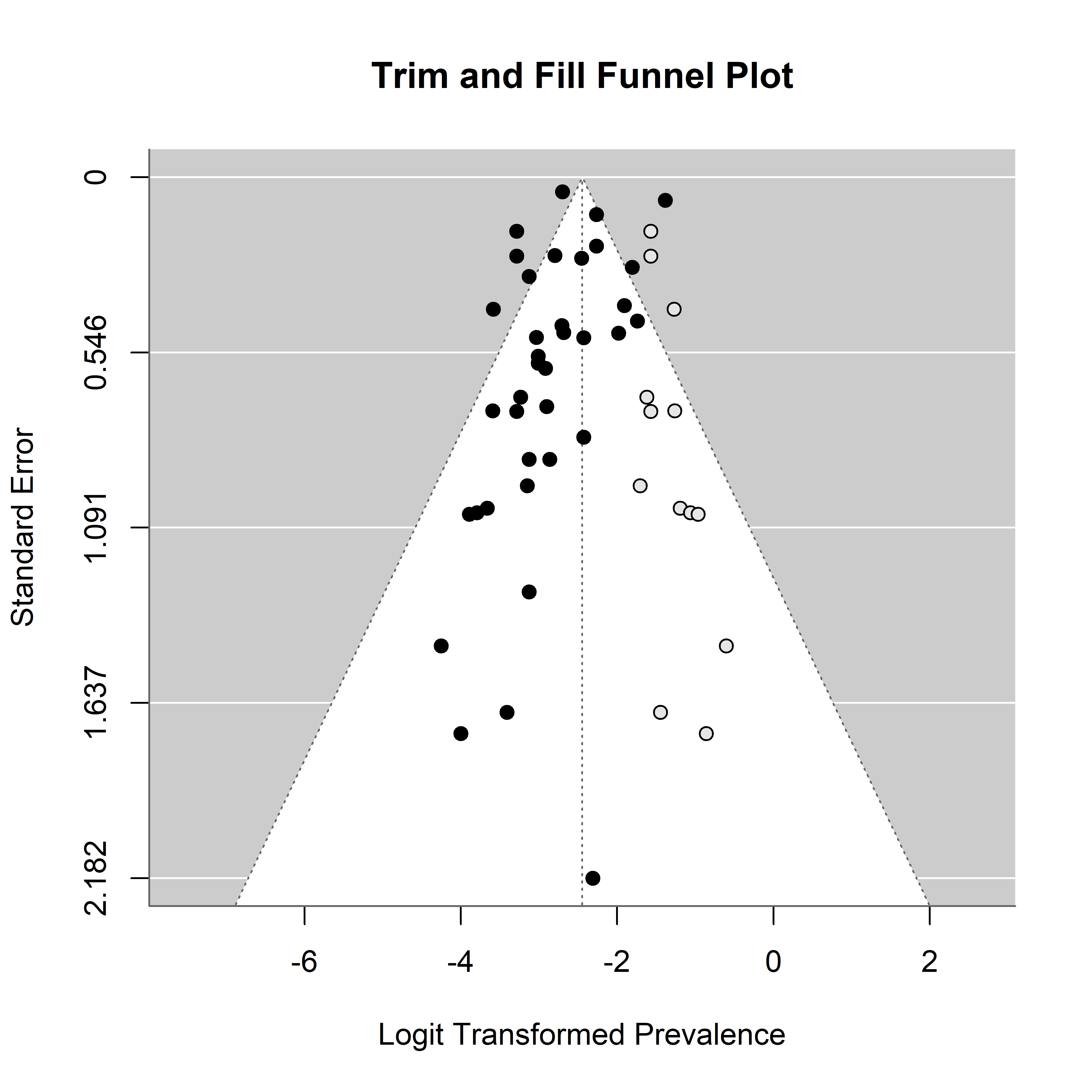

Supplement: Supplementary Materials.docx [file IANN_A_2573143_SM0969.docx]
